# Assembly of the Cardiac Intercalated Disk during Pre- and Postnatal Development of the Human Heart

**DOI:** 10.1371/journal.pone.0094722

**Published:** 2014-04-14

**Authors:** Arnold Vreeker, Leonie van Stuijvenberg, Thomas J. Hund, Peter J. Mohler, Peter G. J. Nikkels, Toon A. B. van Veen

**Affiliations:** 1 Department of Medical Physiology, Division of Heart&Lungs, University Medical Center Utrecht, Utrecht, The Netherlands; 2 Department of Pathology, University Medical Center Utrecht, Utrecht, The Netherlands; 3 The Dorothy M. Davis Heart and Lung Research Institute, The Ohio State University College of Engineering, Department of Biomedical Engineering, The Ohio State University, Columbus, Ohio, United States of America; 4 The Dorothy M. Davis Heart and Lung Research Institute, Department of Physiology & Cell Biology Department of Internal Medicine, Division of Cardiovascular Medicine, The Ohio State University Medical Center, Columbus, Ohio, United States of America; Leiden University Medical Center, Netherlands

## Abstract

**Background:**

In cardiac muscle, the intercalated disk (ID) at the longitudinal cell-edges of cardiomyocytes provides as a macromolecular infrastructure that integrates mechanical and electrical coupling within the heart. Pathophysiological disturbance in composition of this complex is well known to trigger cardiac arrhythmias and pump failure. The mechanisms underlying assembly of this important cellular domain in human heart is currently unknown.

**Methods:**

We collected 18 specimens from individuals that died from non-cardiovascular causes. Age of the specimens ranged from a gestational age of 15 weeks through 11 years postnatal. Immunohistochemical labeling was performed against proteins comprising desmosomes, adherens junctions, the cardiac sodium channel and gap junctions to visualize spatiotemporal alterations in subcellular location of the proteins.

**Results:**

Changes in spatiotemporal localization of the adherens junction proteins (N-cadherin and ZO-1) and desmosomal proteins (plakoglobin, desmoplakin and plakophilin-2) were identical in all subsequent ages studied. After an initial period of diffuse and lateral labelling, all proteins were fully localized in the ID at approximately 1 year after birth. Na_v_1.5 that composes the cardiac sodium channel and the gap junction protein Cx43 follow a similar pattern but their arrival in the ID is detected at (much) later stages (two years for Na_v_1.5 and seven years for Cx43, respectively).

**Conclusion:**

Our data on developmental maturation of the ID in human heart indicate that generation of the mechanical junctions at the ID precedes that of the electrical junctions with a significant difference in time. In addition arrival of the electrical junctions (Nav1.5 and Cx43) is not uniform since sodium channels localize much earlier than gap junction channels.

## Introduction

In the adult human myocardium, an enormous amount of functionally integrated cardiomyocytes form a syncytium that translates electrical activation into contraction of the cardiac muscle. The sequence of events underlying each heartbeat that starts with spontaneous impulse generation in the sino-atrial node and ends with relaxation of the ventricular myocardium, is highly orchestrated. An important aspect that determines functionality of the syncytium is provided by the specific mode through which the individual cardiomyocytes are interconnected. Adult cardiomyocytes are large polarized rod shaped cells with a length of approximately 100 µm and a transversal diameter of about 10 µm. At their longitudinal edges they interconnect via a specialized structure named the intercalated disk (ID). The ID is composed of several interacting protein complexes including intercellular junctions like gap junctions, adherens junctions and desmosomes.

Intercellular mechanical coupling is necessary to assure appropriate cardiac contraction and this is facilitated by the adherens junctions and desmosomes. Adherens junctions are the anchor-point of myofibrils which enable transmission of contractile force from one cell to another [1]. The α-catenin protein of adherens junctions binds to the actin filaments of myofibrils. Subsequently, α-catenin is connected via β-catenin and p120 to the extracellular part, which is mediated by N-cadherin. Desmosomes protect tissues from contractile stress or abrasive forces in cardiac muscle or epithelia. Desmosomes exist of an intercellular region, mediated by desmoglein2 and desmocollin2, and an intracellular region composed of plakoglobin, plakophilin-2 and desmoplakin. The protection from abrasive forces is accomplished by binding of desmoplakin to the intermediate filaments [2]. In adult mammalian cardiomyocytes, the adherens junction and desmosomes seem not spatially separated in distinctive domains but intermingle in a structure called *area composite* [3].

Next to mechanical coupling, gap junctions mediate the intercellular electrical and metabolic coupling through direct communication between neighboring cells. Due to this direct communication ions, small metabolites and signaling molecules can easily move from the cytoplasm of one cell to another. Movement of ions via gap junctions drives electrical impulse propagation in cardiac muscle, which is anisotropic by nature due to the presence of many gap junctions within the ID but relatively few at the lateral cell borders of cardiomyocytes [4]. A gap junction plaque consists of multiple individual channels that are composed of two hemi-channels named connexons that are delivered by the two interconnecting myocytes. In turn, each connexon is composed of a hexamer of connexin proteins.

In addition to the electrical and mechanical junctions, the IDs contain a variety of ion channels that contribute to generation of the electrical impulse. One of them, the voltage-gated cardiac sodium channel Na_v_1.5, is responsible for the rapid upstroke of the cardiac action potential and in that respect, together with the gap junctions, is critically important for maintenance of impulse propagation [5].

In the past decade, the molecular composition and functions of these different ID junctions have been characterized in more detail, and also the maladaptive effects of disturbance of these junctions has been recognized to play an important role in various cardiac pathologies. Many mutations have been described in proteins that compose the junctional structures at the ID, resulting in mislocalization of affected proteins, increased susceptibility to arrhythmias and deterioration of pump function. It is however virtually unknown what the normal sequence of events is during embryonic and postnatal development that stirs the subsequent proteins to locate to the ID. In rats, the spatiotemporal distribution of Connexin43 (Cx43), desmoplakin and N-cadherin, was previously studied by Angst and colleagues [6]. Surprisingly, they showed that, Cx43, desmoplakin and N-cadherin were not fully accumulated in the ID until postnatal day 90. Hypothetically, taking into account a mean life span of rats of 3 years [7], and that in humans of 80 years [8], this would imply that in humans molecular composition of the ID would not be completed before the age of seven years.

In the present study we have been able to study a unique set of 18 human heart specimens ranging in age from 15 weeks in utero (i.u.) to 11 years postnatal. The aim of the study was to follow the changes in distribution of several junctional proteins and Na_v_1.5. Studying this normal development of the IDs in children will add knowledge in order to understand the normal and abnormal electrophysiology of the heart during post-natal development.

## Materials and Methods

### Pediatric cardiac specimens

From the biobank of the Department of Pathology, University Medical Center Utrecht, left ventricular material (middle area of the free wall) was obtained from 18 children who died without any pathological evidence of cardiac involvement. Tissue was made available for research purposes after informed written consent as provided by the relatives in accordance with the institutional guidelines. Specimens raged in age from 15 weeks in utero till 11 years postnatal. Ages, gender and interval till tissue preservation of the 18 individuals are listed in table 1.

**Table 1:** Age of all cardiac specimens that were studied.

Gestational age	Gender	Interval (hr)	Postnatal	Gender	Interval (hr)
15 weeks	m	24–48	1 month	f	12–24
18 weeks	f	<12	5 months	f	<12
19 weeks	f	12–24	10.5 months	m	12–24
22 weeks	f	12–24	1 year 7 months	m	12–24
23 weeks	f	24–48	2 years 5 months	m	<12
30 weeks	f	<12	3 years 6 months	f	12–24
31 weeks	f	12–24	4 years	m	12–24
40 weeks	m	24–48	5 years	f	12–24
			7 years	f	12–24
			11 years	f	12–24

Interval indicates post-mortem period till tissue preservation. m =  male, f =  female.

### Immunofluorescence microscopy

Left ventricular heart samples were cryo-sectioned. Sections of 10 µm thickness were mounted on aminopropyltriethoxysilane (AAS) coated slides and stored at −80 until use. Immunolabeling was performed as described previously [9], and labeled sections were examined using a Nikon Optiphot-2 light microscope equipped for epifluorescence. Digital pictures were taken with a Nikon Digital Sight DS-2MBWc camera.

### Antibodies

The following commercially available antibodies were used: rabbit polyclonal Cx43 (Invitrogen, 1∶250) and mouse monoclonal Cx43 (BD Transduction Laboratories, 1∶200), rabbit polyclonal and mouse monoclonal N-cadherin (both Sigma-Aldrich, 1∶800), mouse monoclonal α-actinin (Sigma-Aldrich, 1∶1000), mouse monoclonal desmoplakin (AbD Serotec, 1∶2000), mouse monoclonal plakoglobin (Sigma-Aldrich, 1∶1000), mouse monoclonal plakophilin-2 (Progen, 1∶1000) and rabbit polyclonal Zona Occludens-1 (ZO-1, Invitrogen, 1∶50). A custom, affinity-purified rabbit anti-human Na_v_1.5 antibody (1∶100) was produced at the Ohio State University, USA [10], [11]. The secondary antibodies donkey anti-mouse DyLight (Dyl, 1∶250), donkey anti-mouse fluorescein (FITC, 1∶100), donkey anti-rabbit TexasRed (TX, 1∶100) and goat anti-rabbit (FITC, 1∶250) were purchased from Jackson Laboratories.

## Results

The aim of this study was to examine developmental changes in subcellular distribution of the junctional proteins and Na_v_1.5, and to identify the time course for localization of the different proteins to the ID. Immunofluorescence microscopy performed on sections of specimens ranging in age from 15 weeks in utero till 11 years postnatal, in general revealed an early movement of mechanical junction proteins from the lateral cell borders of the myocytes towards the ID at the cell termini. Localization to the ID of proteins composing the electrical junctions followed at later time points.

### Mechanical junctions

**Figure 1:**
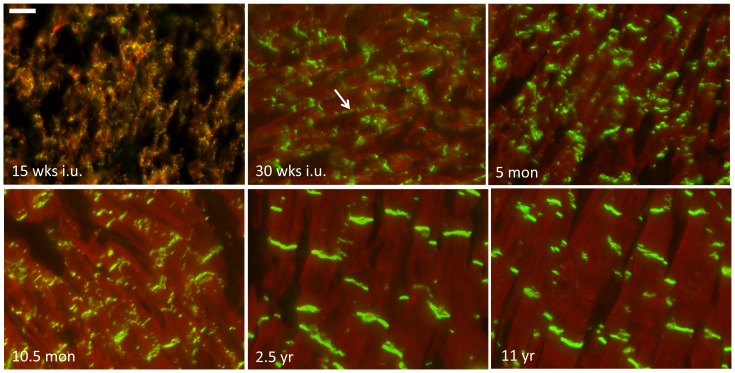
Spatiotemporal organization of N-cadherin. Double labeling of N-cadherin (green) and α-actinin (red) at different stages of cardiac development. Age of the specimen is indicated in the left lower corner of the panels. Scale bar indicates 20 µm.

**Figure 2:**
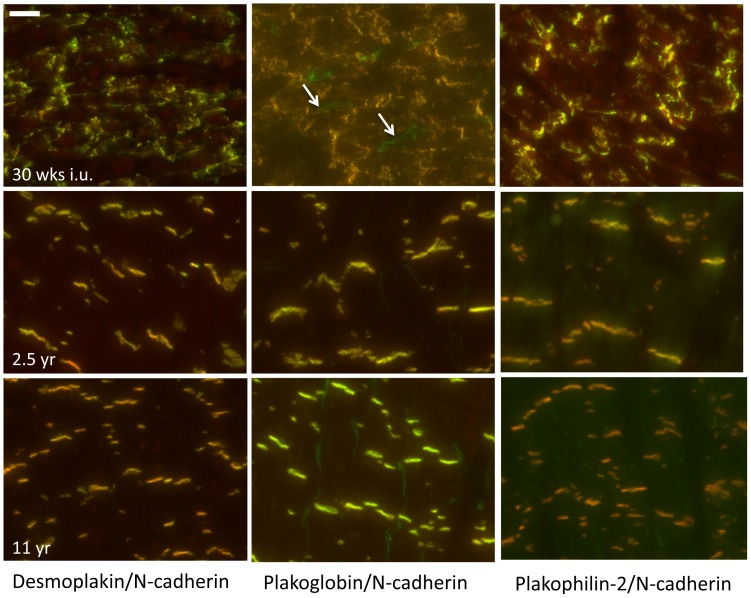
Spatiotemporal organization of desmosomal proteins. During all development stages the desmosomal proteins desmoplakin, plakoglobin and plakophilin-2 (green) colocalize with N-cadherin (red). The plakoglobin signals revealed besides a colocalization with N-cadherin at the IDs also a staining of the capillaries (arrows) between the myocytes. Scale bar indicates 20 µm.

The adherens junction proteins N-cadherin and Zona-Occludens-1 (ZO-1), and the desmosomal proteins desmoplakin, plakoglobin and plakophilin-2 were used to follow the constitution of the mechanical junctions at the ID. Spatiotemporal organization of N-cadherin is shown in Figure 1. At the earliest time-point studied (15 weeks in utero), N-cadherin is merely present as diffuse intracellular signal, which is accompanied by some lateral labelling with spots of higher intensity. As development progresses the diffuse pattern disappears and labeling starts to exist in a mix of lateral labeling and early ID associated signals (Figure 1, 30 weeks i.u.). From that moment N-cadherin progressively moves from the lateral side of the myocytes to the cell termini to form the ID. At the age of 10.5 months postnatal, most N-cadherin has moved to the ID. The images taken from the time-points 2.5 and 11 years fully reflect the pattern seen in adult myocardium. The other mechanical junction proteins, desmoplakin, plakoglobin and plakophilin-2, completely colocalize with N-cadherin during all stages of fetal developmental and postnatal stages (Figure 2). In addition to co-localization with signals for N-cadherin, plakoglobin (Figure 2, 30 weeks i.u., arrows) and ZO-1 (Figure S1B, arrows) labeling also stained capillaries in between the myocytes in all fetal and postnatal stages.

### Electrical junctions and the cardiac sodium channel

**Figure 3:**
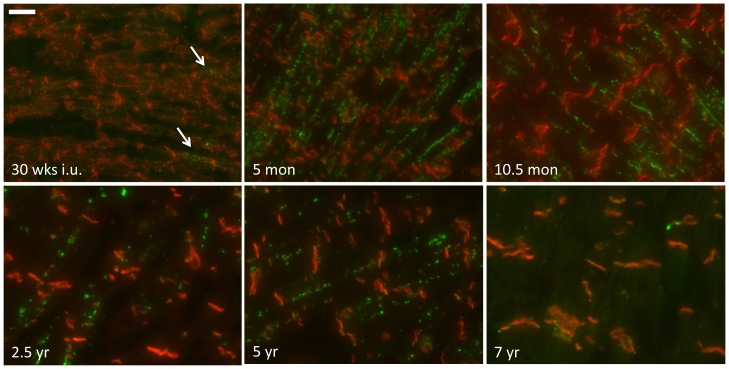
Colocalization of N-cadherin with Cx43. Immunofluorescence of tissue at different stages of cardiac development double-labeled with N-cadherin (red) and Cx43 (green). Scale bar indicates 20 µm.

**Figure 4:**
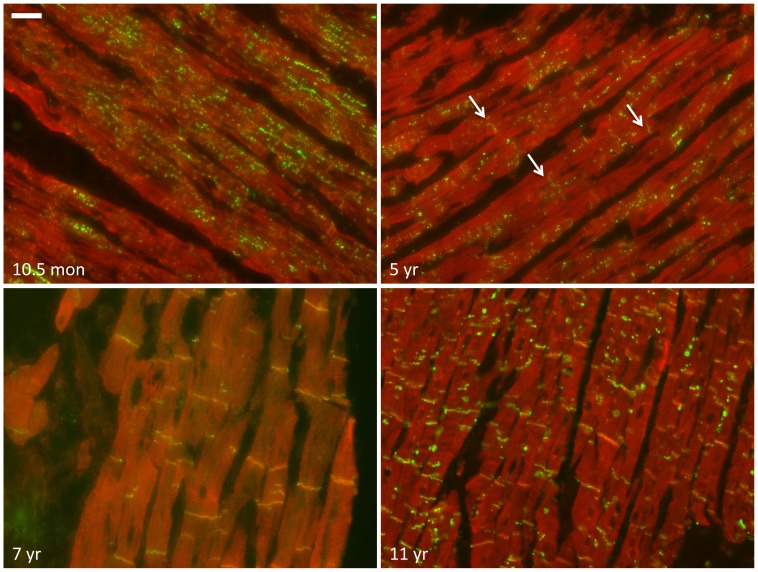
Spatiotemporal movement of Cx43 towards the intercalated disc. Double labeling of Cx43 (green) and α-actinin (red) at different stages of cardiac development. Cx43 (green) Cx43 moves from the lateral side of the myocytes towards the IDs. Arrows indicate less intense ID staining of Cx43 at the age of 5 years when compared to the intensity of lateral signals. Scale bar indicates 40 µm.

Double labeling of the gap junction protein Cx43 and N-cadherin was used to assess their respective subcellular localization during the subsequent stages, and to reveal whether alterations in localization of the electrical junctions followed a similar pattern as observed for the mechanical junctions. Until the developmental stage of 23 weeks in utero, Cx43 protein appeared hardly detectable. From that moment, as illustrated at the stage of 30 weeks in utero in Figure 3 (upper left panel), low amounts of Cx43 (arrows) can be found in a very diffuse pattern without any apparent colocalization with N-cadherin. Around birth, the diffuse presence of Cx43 changes gradually into a pattern of completely lateralized signals (see 5 month postnatal stage in Figure 3 and Figure S1A). At that moment, as already was shown for N-cadherin (Figure 1, upper right panel), the majority of mechanical junctions are already present in the IDs. The lateralization of Cx43 remains present until the stage of five years (Figure 3 and Figure 4 upper panels). In between the postnatal stages 2.5 years and five years, gradually more and more Cx43 can be detected in ID like structures (arrows in Figure 4, upper right panel) although the intensity of immunoreactive signals in these structures is lower when compared to the intensity of lateral signals. In between the age of five and seven years Cx43 is almost completely localized at the ID and shows at seven years (Figure 3, lower right panel) a similar pattern as compared to that at 11 years, the latest time-point studied (Figure 4 lower panels).

**Figure 5:**
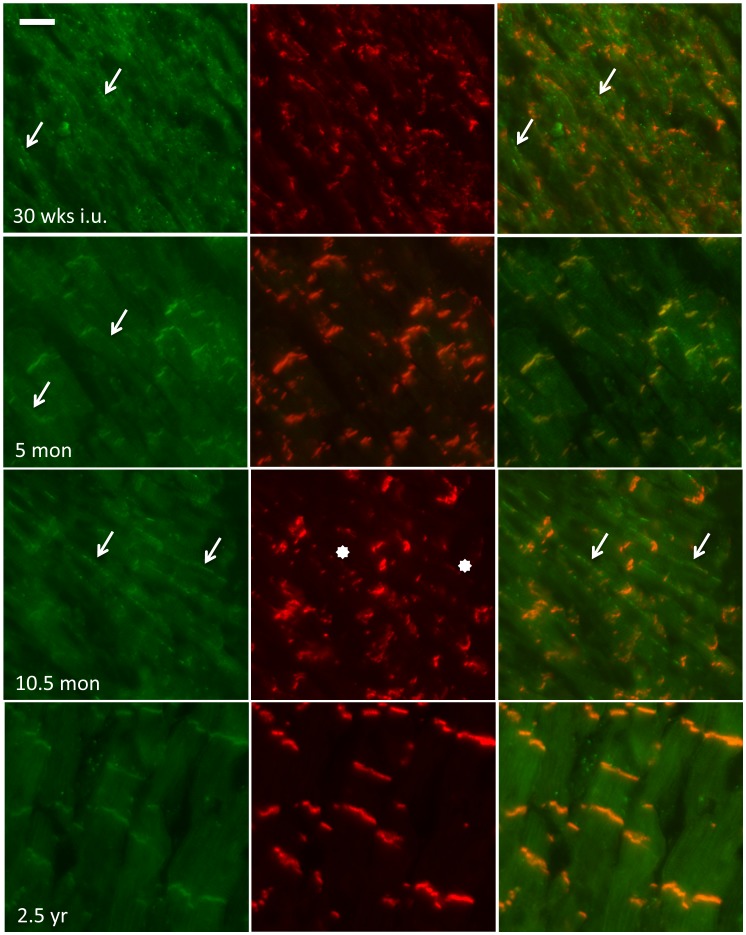
Spatiotemporal movement of Na_v_1.5 towards the intercalated disc. Delayed movement of Na_v_1.5 towards the ID compared to N-cadherin. Until the age of about 1 year ID labeling of Na_v_1.5 is accompanied by lateral labeling (arrows). Stars indicate low abundant N-cadherin signals at these sites. Scale bar indicates 20 µm.

In healthy adult myocardium, the Na_v_1.5 sodium channel, likewise Cx43, shows a colocalization with N-Cadherin at the ID [12]. During development the sodium channel also moves from the lateral sides towards the IDs in a spatiotemporal pattern that is nearly similar to that of N-cadherin. However, during the prenatal stages, early ID association of N-cadherin (Figure 5, 30 wks i.u.) is not yet accompanied by colocalization of Na_v_1.5. In these early stages, Na_v_1.5 is primarily found as diffuse or lateral labelling (arrows). At 5 months postnatal, the most intense signals of Na_v_1.5 can already be found at the ID where they colocalize with N-cadherin (Figure 5, 5 mon). Less intense signals are also found at the lateral sides (arrows). As mentioned (Figure 1), at an age of approximately one year N-cadherin is almost fully organized in the IDs with only some sparse lateral labeling (Figure 5, 10.5 mon, stars). At this timepoint, lateral signals for Na_v_1.5 of more prominent intensity are still present (Figure 5, 10.5 mon, arrows). As shown in Figure 5, lowest panels, Na_v_1.5 is fully located in the IDs at 2.5 years postnatal.

## Discussion

### Main findings

**Figure 6:**
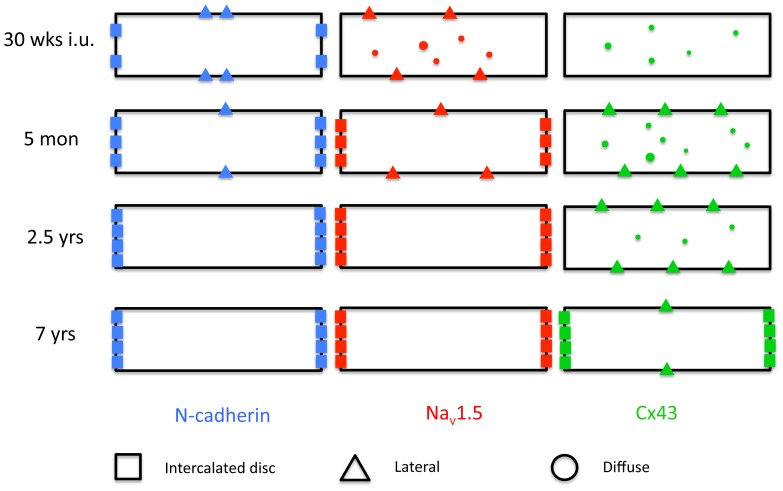
Schematic summary. Cartoon showing a schematic summary of developmental changes in subcellular immunolocalization of N-cadherin (left, blue), Na_v_1.5 (middle, red) and Cx43 (right, green). Black rectangles represent a cardiomyocyte. As pointed out, immunolocalization in cardiomyocytes of plakophilin-2, desmoplakin and plakoglobin is exactly similar to that of N-cadherin at all timepoints.

This study shows that during pre- and postnatal stages of development of the human heart, first the mechanical junctions and the cardiac sodium channel move from the lateral membrane towards the ID which is followed much later in time by the gap junction protein Cx43. All the mechanical junction proteins studied colocalize with N-cadherin and, as such, follow the same spatiotemporal changes in organization. Figure 6 shows a schematic summary of the findings in this study.

### Establishment of the mechanical junctions

After approximately one year postnatal, almost all mechanical junctions are established in the IDs and show a pattern comparable to adults. This suggests that in children under the age of two years the myocardial syncytium may be less integrated, which might give rise to an impaired contractility compared to adult heart. Friedman *et al*. studied the extent of shortening and velocity of shortening in relation to the developed tension. They showed a slower contraction velocity in fetal hearts compared to adult hearts and suggested that one of the causative reasons could be a lower number of contractile units or sarcomeres in young hearts [13]. Our data might add an additional reason since lateral expression of mechanical junctions rather than an ID-associated profile fits well to this impaired contraction velocity. Various studies also suggest a similar pattern of spatiotemporal alterations of junctional proteins that localize to the ID both *in vitro* [14]–[16], and *in vivo* [6], [17]. Using rat neonatal cardiomyocytes either cultured in 2D or 3D (in matrigel), Zhou and colleagues showed that both desmosomal (plakoglobin, plakophilin-2) and adherens junction proteins (N-cadherin) preceded gap junction proteins (Cx43) in formation of new intercellular contacts, comparable as was shown in intact rat heart [6]. Data from these studies, and from the present study are partially in line with data presented two decades ago by Peters *et al* [17]. In their study they analyzed human ventricular specimens that ranged in age from 4 weeks postnatal till 15 years. Although their conclusion that Cx43 is fully localized to the ID at the age of 6 years is in line with our conclusion, they describe that the transitional changes in localization of N-cadherin and Cx43 are similar in space and time. This observation contrast to that in the aforementioned studies, and to our data, for which we do not have an explanation.

### Expression of Cx43 and Na_v_1.5 during development

Together with the cardiac sodium channel, Na_v_1.5, gap junctions facilitate a fast propagation of the electrical impulse. In adult heart, predominant localization of gap junctions at the ID gives rise to a fast longitudinal velocity along with the fiber orientation, whereas conduction velocity perpendicular to the fiber orientation is much slower. Our data show that only after an age of seven years Cx43 is fully organized in the IDs. In children younger than five years Cx43 is predominately expressed at the lateral membranes while in the developmental stages before birth Cx43 appeared present only in negligible amounts in a diffuse pattern. Still hearts of newborns contract normally and beat in general in a perfect sinus rhythm. It is known that a large safety margin exists when it comes to intercellular coupling and appropriate conduction [18]. It is also known that during developmental stages of the rodent heart two other gap junction isoforms (Cx40 and Cx45) can be expressed by ventricular cardiomyocytes [19], [20]. These isoforms form in the adult stage the gap junctions within the specialized cardiac conduction system [21]. In our specimen, labeling against Cx40 did not reveal intercellular signals between the cardiomyocytes rather than positive signals only in the endothelium of the vessels (data not shown). Unfortunately we did not have access to antibodies against Cx45 that were specific enough to investigate whether this isoform adds to gap junction formation at the human ID in the developmental stages before birth.

Electrophysiological studies on adult and neonatal dog hearts showed a significant slower transversal conduction velocity of the action potential in adult heart compared to neonatal. In neonates there was no difference in transversal and longitudinal propagation [22], and the same findings were described in cultures of rat neonatal cardiomyocytes [23]. In the same canine model immunohistological experiments showed lateralized expression of Cx43 in hearts ranging in age from 1 day to 2 months postnatal, whereas in adult hearts an exclusive ID staining was demonstrated. These experimental data clearly indicate a strong relation between developmental changes in the distribution of ventricular gap junctions and the characteristics of electrical impulse propagation. In all probability, a human newborn likely shows the same isotropic conduction characteristics as those described in the canine model [22]. Likely the predominant expression of Cx43 at the lateral sides in combination with the rather robust expression of Nav1.5 in the ID as found in our study, could be an explanation for isotropic conduction. Similarly, the shift in localization of Cx43 from the lateral sides towards the ID fits well with the changes from isotropic conduction towards anisotropic conduction.

The distribution of gap junctions at the lateral side of the cardiomyocytes might possibly have a metabolic function as well. During development, changes in cardiac metabolism occur. In rabbit fetal and immediate newborn hearts glycolysis is the main contributor to cardiac ATP production [24]. However, during progressive maturation of the neonate the contribution of fatty acids in mitochondrial oxidative metabolism increases and becomes the major contributor to ATP production in adult cardiomyocytes. These changes occur due to changes in transcription of HIF-1α, PGC-1α/PPARα and PGC-1α/PPARδ [24]. To speculate, during development the lateral organized gap junctions are possibly able to contribute to the transport of glucose for the fetal and neonatal ATP production. Therefore a functional interaction may exist between the changes in cardiac metabolism and the changes in the distribution of gap junctions.

The fast anisotropic conduction through the IDs in mature myocardium is supported by the organization of Na_v_1.5 sodium channels at the IDs. As this study shows, until an age of two years Na_v_1.5 is localized at the ID as well as, though less prominent, at the lateral side of the cardiomyocytes. Several studies have shown for different species an exclusive staining of Na_v_1.5 at the IDs while others show additional staining at the lateral membranes [25]–[27]. These contradictory findings might partially be due to the differences in age of the animals and human specimen studied, and the fact that, sometimes, isolated cells were used for the experiments. In addition it can not be ignored that some of the antibodies used in these studies recognized both the Na_v_1.5 cardiac sodium channels as well as the ‘brain type sodium channels’ that form a minority in cardiac myocytes and seem specifically functional at the lateral membranes [28], [29]. That Na_v_1.5 is both present in the ID and at the lateral membrane can be defended by the expression pattern of syntrophin-dystrophin complex and SAP97 which influence Na_v_1.5 trafficking. The syntrophin-dystrophin complex interacts with Na_v_1.5 at the lateral border and similar does SAP97 at the ID [30], [31].

### Concluding remarks

The obvious question that remains to be answered is of course what mechanisms stir the formation of mature IDs and the spatiotemporal movement of the different proteins that compose the ID. The answer to this question will require additional knowledge regarding the identity and regulation of macromolecular protein complexes at the ID. It is likely that several components that are of importance have not been identified yet. The composition of the ID has recently been reviewed thoroughly by Wang et al [32]. Next to a description of the networks that are known and the alterations that take place in the complexes during disease or artificial modulation of individual components in genetically engineered models, they also touch upon ID formation and propose a leading role for Xin proteins [33]. Xin proteins could be leading in the regulation of ID targeting by creating complexes with N-cadherin, β-catenin and p120-catenin [34]. Besides the catenins and N-cadherin, plakophilin-2 fulfills a crucial role in maintaining the composition of the *area composita* [35]. Interestingly, previous studies have shown that plakophilin-2 likely forms a macromolecular complex in which both Cx43 and Na_v_1.5 are included [36]–[38]. Moreover, loss of Cx43 at the ID results in a reduction of Na_v_1.5 and the accompanying sodium current [39]. If plakophilin-2, Cx43 and Na_v_1.5 are co-regulated within the ID, this raises the question whether the mechanism that regulates this is similar under mature conditions and during the developmental stages. Given the fact that our data show that the appearance of Na_v_1.5 in the ID is much earlier than that of Cx43, this suggest that the answer is no. It might well be that during maturation parts of the molecular puzzle are initially lacking to fulfill their required function and the temptation is to identify these entities. With that knowledge we hopefully will be able to understand ID formation in more detail and this information might also be applicable to a better understanding of the molecular changes at the ID during cardiac disease.

## Study Limitations

In this study we have examined a unique set of left ventricular human specimens that ranged from a gestational age of 15 weeks to a postnatal age of 11 years. Despite the unique nature of the available tissue, some considerations have to be mentioned with respect to the executed experimental approach and the interpretation of acquired data. Each specimen studied was of a different age and no biological replicates were available. This means that the aspect of inter-individual differences (at each time-point) could not be included. Because of that, also potential gender related differences in assembly of the ID can't be excluded. Since all specimens were of left ventricular origin, potential differences in assembly between e.g. the right and left ventricle were not studied. Finally, the specimens have been collected at autopsy following a highly standardized procedure using a design with special attention to prevent as much as possible confounding post mortal damage to the tissue. Although every individual specimen has been examined thoroughly for the localization of all mentioned proteins in this study, we can not completely exclude any influence regarding the aspect of time between death and autopsy.

## Supporting Information

Figure S1 A: enlarged magnification of lateral Cx43 signals (green) and predominant ID signals for N-cadherin (red) at the age of 5 months. B: The adherens junction protein ZO-1 colocalizes with N-cadherin during the fetal development and postnatal. N-cadherin (red) and ZO-1 (green). In all stages ZO-1 labeling additionally showed capillaries between the myocytes (arrows). Scale bar indicates 20 µm in A and 40 µm in B.
(TIF)
