# Supplementary figures and images for: Assembly of the Cardiac Intercalated Disk during Pre- and Postnatal Development of the Human Heart
Source: PLoS One. 2014 Apr 14;9(4):e94722. doi: 10.1371/journal.pone.0094722 (PMC3986238; doi:10.1371/journal.pone.0094722)

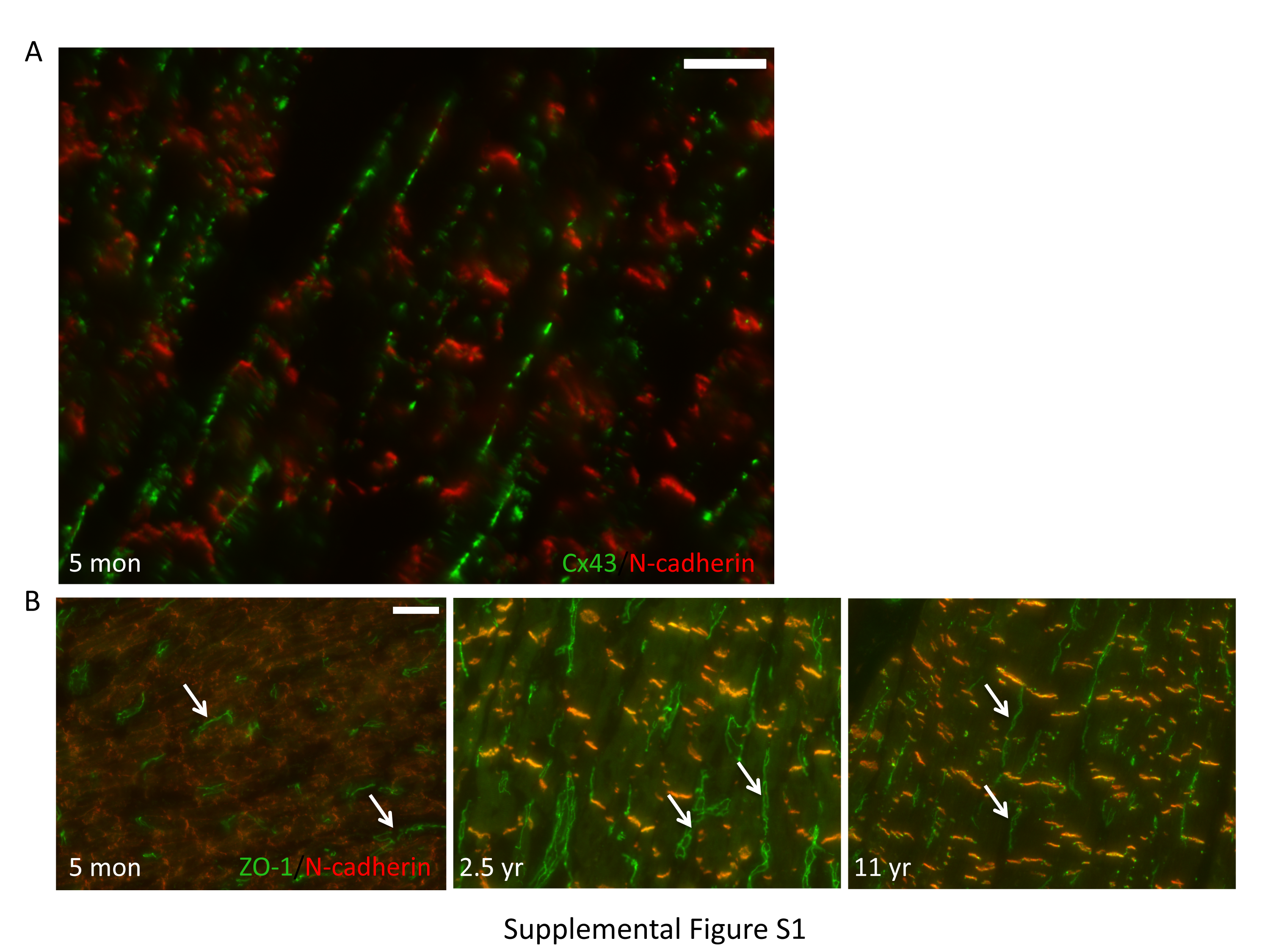

Supplement: Figure S1 — A: enlarged magnification of lateral Cx43 signals (green) and predominant ID signals for N-cadherin (red) at the age of 5 months. B: The adherens junction protein ZO-1 colocalizes with N-cadherin during the fetal development and postnatal. N-cadherin (red) and ZO-1 (green). In all stages ZO-1 labeling additionally showed capillaries between the myocytes (arrows). Scale bar indicates 20 µm in A and 40 µm in B. (TIF) [file pone.0094722.s001.tif]
